# Development and Use of a Residence Time Distribution (RTD) Model Control Strategy for a Continuous Manufacturing Drug Product Pharmaceutical Process

**DOI:** 10.3390/pharmaceutics14020355

**Published:** 2022-02-03

**Authors:** Samantha Hurley, Anthony Tantuccio, Manuel Sebastian Escotet-Espinoza, Matthew Flamm, Matthew Metzger

**Affiliations:** 1 Pharmaceutical Commercialization Technology, Merck & Co., Inc., West Point, PA 19486, USA; anthony.tantuccio@merck.com (A.T.); matthew.metzger@merck.com (M.M.); 2 Pharmaceutical Sciences, Merck & Co., Inc., Rahway, NJ 07065, USA; manuel.escotet.espinoza@merck.com; 3 Applied Mathematics and Modeling, Merck & Co., Inc., West Point, PA 19486, USA; matthew.flamm@merck.com

**Keywords:** continuous manufacturing, continuous direct compression, residence time distribution, control strategy

## Abstract

Residence-time-distribution (RTD)-based models are key to understanding the mixing dynamics of continuous manufacturing systems. Such models can allow for material traceability throughout the process and can provide the ability for removal of non-conforming material from the finished product. These models have been implemented in continuous pharmaceutical manufacturing mainly for monitoring purposes, not as an integral part of the control strategy and in-process specifications. This paper discusses the steps taken to develop an RTD model design space and how the model was statistically incorporated into the product’s control strategy. To develop the model, experiments were conducted at a range of blender impeller speeds and total system mass flow rates. RTD parameters were optimized for each condition tested using a tank-in-series-type model with a delay. Using the experimental RTD parameters, an equation was derived relating the mean residence time to the operating conditions (i.e., blender impeller speed and mass flow rate). The RTD parameters were used in combination with real-time upstream process data to predict downstream API concentration, where these predictions allowed validation across the entire operating range of the process by comparison to measured tablet assay. The standard in-process control limits for the product were statistically tightened using the validation acceptance criteria. Ultimately, this model and strategy were accepted by regulatory authorities.

## 1. Introduction

Traditional industrial processing, such as petrochemical, steel, and high-volume food manufacturing, is performed using continuous processes. Continuous processes are a shift from the original batch processes, which mainly occurred in order to reap the many benefits of continuous manufacturing [1,2,3]. Benefits of continuous processes include reduced cost and processing time and improved ability to implement time-independent control strategies [4,5]. Within the pharmaceutical industry, this shift has only started to occur within the last few years due to many regulatory constraints and a relatively risk-averse culture. The push for the change in the pharmaceutical industry is due to the potential improvements to product flow in supply chains, such as leveraging fast cycle times and flexible batch sizes, and to a reduction in lead times and inventory levels [6,7,8]. Continuous drug substance and product manufacturing has been sparked by the FDA as they continually express their support for the development and implementation of continuous manufacturing for drug product processing [9,10,11].

A major hurdle for the pharmaceutical industry in its shift towards continuous manufacturing involves the regulatory requirement regarding the ability to ensure tight control of active ingredients in products (i.e., tracking of materials through the system) [10,12,13]. Typical continuous manufacturing control strategies in the industry have heavily leveraged the use of NIR spectroscopy for blend composition monitoring [14]. Alternate methods for downstream monitoring include the use of residence time distribution (RTD) modeling [6]. Such models have typically been used to describe non-ideal fluid flow [15]. RTD models have been applied successfully to powder processing, such as twin screw co-rotating mixers, circulating fluidized beds, and continuous horizontal blenders [6,16,17]. 

A robust control strategy can be developed around a fully characterized RTD as a part of a design space [18]. Typical characterization of RTD models within a design space establishes an understanding of the relationship between operating conditions and the mean residence time of the system [15]. Measurement techniques for characterization include impulse experiments or step change experiments, where the concentration of a specific material is intentionally varied [6,19]. RTD optimization may include models such as axial dispersion or tanks-in-series and leverage various convolution techniques [15]. Regardless of the optimization technique, a characterized RTD model can allow for real-time prediction of active pharmaceutical ingredient (API) concentration downstream as a result of upstream disturbances [6]. 

The first part of this work defines the RTD model structure and explores the relationship of mass flow and blender impeller speed to the mean residence time of the system. It establishes the relevant assumptions and conditions. The second part of this paper summarizes the validation strategy and results. Both topics outline the successful development and application of an RTD model for powder processing in the pharmaceutical industry. 

## 2. Materials and Methods

### 2.1. Formulation and Process

**Figure 1:**
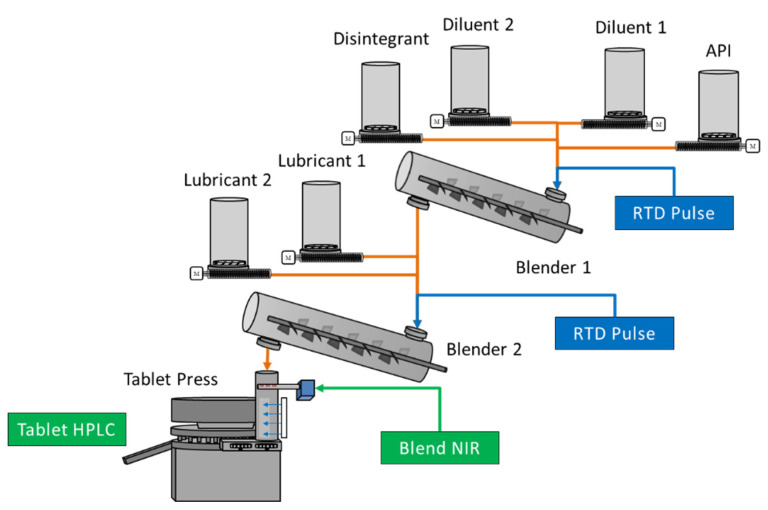
Process flow diagram of the GEA CDC-50 Rig.

All experiments were conducted using the GEA CDC-50 rig [20]. The equipment is composed of six loss-in-weight (LIW) feeders. The formulation used for these experiments involves a commercial formulation composed of an active pharmaceutical ingredient (API), two diluents, a disintegrant, and two lubricants. Given the commercial nature of the product, the qualitative composition of the product will be described. The API comprises more than 25% of the formulation composition by weight. The diluents in the system are microcrystalline cellulose and anhydrous dicalcium phosphate. The disintegrant and lubricants are croscarmellose sodium, magnesium stearate, and sodium stearyl fumarate, respectively. The API, diluents, and disintegrant are fed into a continuous linear blender, denoted as Blender 1, and comprise 96% of the formulation. The outlet of the first blender is fed into a second continuous linear blender, denoted as Blender 2, along with the two lubricants, which comprise the remaining 4% of the formulation. The outlet of the second blender is fed past a blend NIR unit and then into a rotary tablet press [21]. In this process the API concentration was measured in two distinct areas: (1) the blend, via the blend NIR unit at the outlet of Blender 2, and (2) the tablets, via tablet HPLC. Development of these two methods is further discussed in Section 2.4. The overall equipment and process design are described in Figure 1.

#### RTD and Impulse Experiments

A widely used method of RTD characterization for a CM system is performing impulse experiments. Impulse experiments are performed by adding an instantaneous and precise quantity of a tracer into the inlet of a process, while the change in the tracer concentration at the outlet of the process is measured over time [4,22]. For the purpose of this work, impulse experiments were performed by introducing an API tracer independently into Blender 1 and Blender 2 to evaluate the downstream response of API concentration measured by blend NIR and/or tablet assay. In Figure 1, these impulse introduction locations are indicated in blue, and the measurement locations are indicated in green. Work was performed with various tracer quantities to establish the appropriate amount: (1) to enable sufficient detection by the measurement methods, and (2) to not alter the flow of powder in the process. These quantities were 30 g of API for Blender 2 and 100 g of API for Blender 1 impulse experiments, and these tracer quantities were leveraged throughout these experiments.

**Figure 2:**
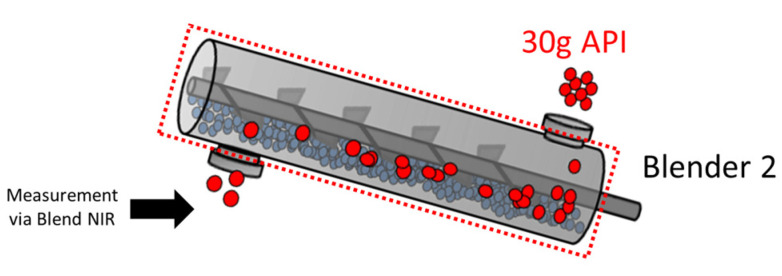
Graphical Representation of a Blender 2 Impulse Experiment.

Because of the in-series nature of the blenders, experiments to characterize the mixing had to be performed in reverse order, meaning characterization of Blender 2 preceded the characterization of Blender 1. To determine the RTD behavior in Blender 2, 30 g of API was introduced into the inlet of the blender, and the concentration data were collected at the outlet of Blender 2. A graphical representation of these experiments is shown in Figure 2. In Blender 2 impulse experiments, the change in API concentration was measured via the blend NIR unit and can be isolated to Blender 2, as indicated by the dotted red line around the process in Figure 2.

**Figure 3:**
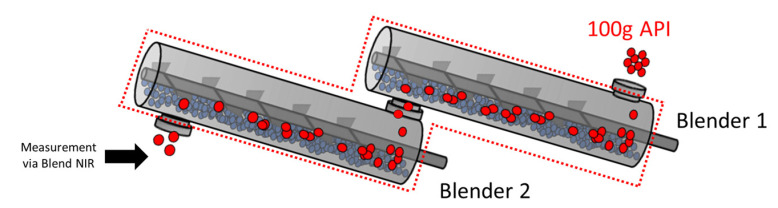
Graphical Representation of a Blender 1 Impulse Experiment.

To determine the RTD behavior in Blender 1, 100 g of API was introduced into the inlet of the blender, and concentration data were collected at the outlet of Blender 2. A graphical representation of these experiments is shown in Figure 3. Due to the location of the blend NIR unit, the Blender 1 experiments effectively captured the change in concentration in both Blender 1 and 2, as indicated by the dotted red line around the process in Figure 3. The data from these experiments were used in combination with the Blender 2 impulse data to isolate the RTD behavior for Blender 1. The method for isolation is described in Section 2.3.1.

In addition to the blend NIR data collected during the Blender 1 impulse experiments, tablet samples were collected approximately every 20–30 s. These tablet samples were tested for API concentration via HPLC. These data were used in combination with both the Blender 1 and 2 impulse data to isolate the RTD behavior for the feed frame, as no impulse experiments were performed directly into the feed frame. The isolation procedures are further described in Section 2.3.1.

The concentration of the API, measured as the function of time C(t), for all experiments was transformed to the probability distribution function E(t) using Equation (1) [23].

(1) $$E\left(t\right)=\frac{C\left(t\right)}{\int_{0}^{\infty }C\left(t\right)dt}$$

The mean residence time (*MRT*) of a system is defined as the average time particles spend in the system. Mathematically, the *MRT* is defined as in Equation (2) [24,25].

(2) $$MRT=\int_{0}^{\infty }tE\left(t\right)dt$$

### 2.2. RTD Model Implementation and Optimization

#### 2.2.1. Model Mathematical Structure

The CDC-50 is comprised of three independent blending units: Blender 1, Blender 2, and the tablet press feed frame. The CDC-50 utilizes an RTD-based process model that defines each blending unit as the combination of two theoretical mixing systems: a fully segregated system and a well- or ideally mixed system. The segregated mixing system is described in the literature as a plug flow reactor (PFR), while the well-mixed system is known as a continuous stirred tank reactor (CSTR). Notably, these labels describe the mixing systems as reactors, but no reaction is occurring in these systems. The system’s mathematically described mixing behavior is the model being represented in this work.

**Figure 4:**
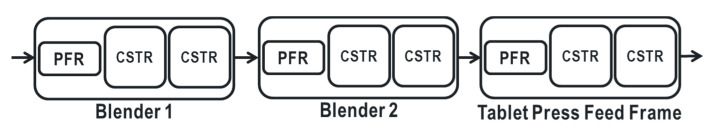
Schematic of Blending Unit and Theoretical Reactor Design.

Each of the mixing units in the CDC-50 is modeled as a single PFR followed by two CSTRs, as shown in Figure 4. This model structure is what GEA has previously defined as the flow used in their CDC-50 [21]. Additionally, this model structure was found to sufficiently describe the blending capability of the system while minimizing the number of regressed parameters. However, these theoretical reactors are a mathematical representation of the blending capability of the system, and do not have a direct physical meaning for the process.

For an ideal PFR, all particles have spent the same amount of time in the reactor. The RTD for a PFR can be mathematically described by Equation (3):

(3) $$E_{plug}\left(t\right)=\delta \left(t-\theta \right)$$

where δ is the Dirac delta function, θ is the mean residence time of the PFR, or more simply the delay time, and *t* is time [26]. In an ideal CSTR, the effluent concentration is the same as the concentration throughout the reactor, and there is perfect and instantaneous mixing. The RTD for a CSTR can be described by Equation (4):

(4) $$E_{tank}\left(t\right)=\frac{e^{-t/\tau_{tank}}}{\tau_{tank}}$$

where τ is the mean residence time of the CSTR, and t is time [26]. The RTD model of each blending unit is the mathematical convolution of all three theoretical reactors in that unit, and the convolution is described in Equation (5) [27]:

(5) $$E_{1}*E_{2}=E_{1*2}=\int_{0}^{t}E_{1}\left(x\right)\cdot E_{2}\left(t-x\right)dx$$

where $E_{1}$ and $E_{2}$ are RTD equations for any reactor 1 and 2, respectively. Applying the convolution in Equation (5) to the combination of each reactor for any given unit operation (e.g., Blender 1, Blender 2, or feed frame) leads to the 2-CSTR-in-series model with a delay. The equation for this model is Equation (6).

(6) $$E{\left(t\right)}_{Unit}=\frac{Exp\left[-\frac{t-\theta }{\tau_{tank,1}}\right]-Exp\left[-\frac{t-\theta }{\tau_{tank,2}}\right]}{\tau_{tank,\;1}-\tau_{tank,\;2}}$$

Parametrically, this equation has three regressed coefficients: $\tau_{tank,\;1}$, $\tau_{tank,\;2}$, and θ. Note that the two tank sizes are non-unique variables, which may lead to uncertainty and high non-linearity in the optimization. To prevent the non-unique solutions, an additional constraint must be placed in the model where $\tau_{tank,\;1}>\tau_{tank,\;2}>0$. This constraint further avoids undetermined or negative solutions. The additional constraint can lead to high standard errors of regressions for the parameters, which provides lower confidence for this model. Thus, the model was modified for our analysis by making the two tanks the same size ($\tau_{tank,\;1}=\tau_{tank,\;2})$. Using the assumption of equal tank size yields the generalized 2-CSTR-in-series model with a delay described in [19] for all future analysis. This equation is Equation (7) where the regressed coefficients are $\tau_{tank}$ and θ.

(7) $$E{\left(t\right)}_{Unit}=\frac{\left(t-\theta \right)\;Exp\left[-\frac{t-\theta }{\tau_{tank}}\right]}{\tau_{tank}{}^{2}}$$

Based on the description in Figure 4, there are three different models for the system. For each individual blending unit, the model matches what is shown in Equation (7), and the models are summarized in Equations (8)–(10) for Blender 1, Blender 2, and the feed frame, respectively.

(8) $$E{\left(t\right)}_{B1}=\frac{\left(t-\theta_{B1}\right)\;Exp\left[-\frac{t-\theta_{B1}}{\tau_{tank,B1}}\right]}{\tau_{tank,B1}{}^{2}}$$

(9) $$E{\left(t\right)}_{B2}=\frac{\left(t-\theta_{B2}\right)\;Exp\left[-\frac{t-\theta_{B2}}{\tau_{tank,B2}}\right]}{\tau_{tank,B2}{}^{2}}$$

(10) $$E{\left(t\right)}_{FF}=\frac{\left(t-\theta_{FF}\right)\;Exp\left[-\frac{t-\theta_{FF}}{\tau_{tank,FF}}\right]}{\tau_{tank,FF}{}^{2}}$$

Similar to Equation (7), Equations (8)–(10) yield the regressed coefficients $\tau_{tank,B1}$ and $\theta_{B1}$ for Blender 1, $\tau_{tank,B2}$ and $\theta_{B2}$ for Blender 2, and $\tau_{tank,FF}$ and $\theta_{FF}$ for the feed frame.

#### 2.2.2. Mathematical Model Optimization 

The resulting API concentration of the blend collected via the blend NIR unit during Blender 1 impulse experiments contains the parameters for both blenders, B1 + B2 (e.g., $E{\left(t\right)}_{B1+B2}$). Thus, the model for Blender 1 impulse experiments is the convolution of the Blender 1 and Blender 2 models, as summarized in Equation (11).

(11) $$E{\left(t\right)}_{B1+B2}=E{\left(t\right)}_{B1}*E{\left(t\right)}_{B2}$$

In addition, the API concentration of the tablets collected via HPLC during the Blender 1 impulse experiments contains the parameters for the entire system, B1 + B2 + FF (e.g., $E{\left(t\right)}_{system}$), as summarized in Equation (12).

(12) $$E{\left(t\right)}_{system}=E{\left(t\right)}_{B1+B2}*E{\left(t\right)}_{FF}$$

Since each blending unit is the mathematical combination of the three reactors, the total mean residence time (*MRT*) of a blending unit can be defined as the sum of each individual reactor residence time as described in Equation (13).

(13) $$MRT_{Unit}=\theta +2\tau_{tank}$$

Preliminary observations pointed to a trend between $\tau_{tank}$ and θ across all operating conditions within a blending unit where the ratio of $\tau_{tank}$ to θ was relatively consistent. This observed trend was utilized to further constrain the optimization. Therefore, $\frac{\tau_{tank}}{\theta }$ is defined as a constant value across all operating conditions within a blending unit. This constant will be further described as *R* (i.e., $R=\frac{\tau_{tank}}{\theta }$), and each blending unit will have a single *R* value, yielding regressed values for *R*_*B*1_, *R*_*B*2_, and *R_FF_* in addition to $\tau_{tank,B1}$ and $\theta_{B1}$ for Blender 1, $\tau_{tank,B2}$ and $\theta_{B2}$ for Blender 2, and $\tau_{tank,FF}$ and $\theta_{FF}$ for the feed frame. Using the combination of *R* and Equation (11), the *MRT* can be defined as Equation (14).

(14) $$MRT_{Unit}=\tau_{tank}\left(2+\frac{1}{R}\right)$$

Equations (11) and (14) also allow direct calculation of $\tau_{tank}$ to θ if the *MRT* value is known, which is further discussed in Section 4.2. In addition to the regressed parameters, a “goodness of fit statistic—ε” of the model optimization was generated for each $\tau_{tank}$ and *R* regressed value. These ε values were used to generate a goodness of fit of the mean residence time (*MRT*) using the propagation of error. Using Equation (14) and partial fractions, the goodness of fit for *MRT* ($\varepsilon_{MRT}$) is described in Equation (15). This statistic was used to assess how well the optimization method was able to predict the *MRT* and was used as weighting factor in further analysis discussed in Section 3.

(15) $$\varepsilon_{MRT}{}^{2}={\left(2+\frac{1}{R}\right)}^{2}\varepsilon_{\tau_{tank}}{}^{2}+{\left(-\frac{\tau_{tank}}{R^{2}}\right)}^{2}\varepsilon_{R}{}^{2}$$

### 2.3. Experimental Design and Optimization

**Figure 5:**
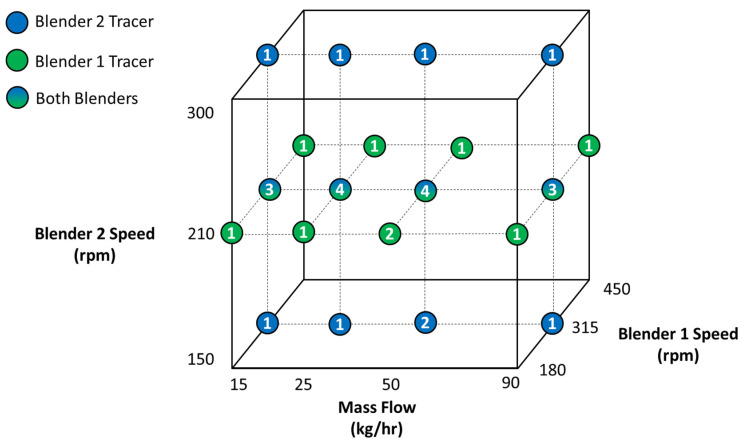
Summary of RTD Impulse Experiments.

Previous studies have shown that blender impeller speed and mass flow rate have an impact on the RTD of a CM system [24,28,29,30]. Therefore, during these experiments, three major variables were studied at varying levels: impeller speed for Blender 1, impeller speed for Blender 2, and total line mass flow rate. Impulse experiments were conducted at total system mass flow rates between 15–90 kg/h. Blender 1 and Blender 2 impeller speeds were operated at 180–450 rpm and 150–300 rpm, respectively. A summary of all conditions tested is given in Figure 5, where the numbers indicate the number of replicates completed at each condition. The blue circles indicate that only Blender 1 impulse experiments were conducted, green circles indicate that only Blender 2 impulse experiments were conducted, and the multi-colored circles indicate that both Blender 1 and 2 impulse experiments were conducted. The tablet press turret speed was specified by the total system mass flow rate and the mass of each tablet unit. The feed frame paddle speed was not explored as a factor during these experiments. The center points for both blenders at each mass flow rate were used to isolate the feed frame behavior. 

#### 2.3.1. Experimental Model Optimization

A non-linear least-squares regression was developed in MATLAB using Equations (8)–(12) and was executed using the experimental Blender 2 impulse *E*(*t*) data collected at each of the operating conditions summarized in Figure 5. *τ*_*tank*,*B*2,*i*_ was simultaneously regressed across all 12 conditions, while also regressing *R*_*B*2_. Additionally, each *τ*_*tank*,*B*2,*i*_ and *R*_*B*2_ value had a corresponding “goodness of fit—ε” value. In total, 25 Blender 2 parameters were regressed. 

Since the *E*(*t*) data collected during Blender 1 impulse experiments included the mixing behavior for both Blender 1 and Blender 2, the *τ*_*tank*,*B*2,*i*_ and *R*_*B*2_ were used to deconvolute the model response during optimization of Blender 1 parameters. At the corresponding conditions for Blender 1 impulse experiments, *τ*_*tank*,*B*2,*i*_ was input as a known parameter. A similar non-linear least-squares regression was executed in MATLAB using the experimental Blender 1 impulse *E*(*t*) data collected at each of the operating conditions summarized in Figure 5. *τ*_*tank*,*B*1,*i*_ was simultaneously regressed across all 12 conditions, while also regressing *R*_*B*1_. Additionally, each *τ*_*tank*,*B*1,*i*_ and *R*_*B*1_ value had a corresponding “goodness of fit—ε” value. In total, 25 Blender 1 parameters were regressed.

Similar to Blender 1, the tablet press feed frame response included mixing behavior from Blender 1, Blender 2, and the feed frame. Therefore, *τ*_*tank*,*B*1,*i*_, *R*_*B*1_, *τ*_*tank*,*B*2,*i*_, and *R*_*B*2_ results were used to deconvolute the model response during optimization of the feed frame parameters. At the corresponding conditions for Blender 1 and Blender 2 impulse experiments, *τ*_*tank*,*B*1,*i*_ and *τ*_*tank*,*B*2,*i*_ were input as known parameters. A similar non-linear least-squares regression was executed in MATLAB using the experimental feed frame impulse *E*(*t*) derived from tablet samples during Blender 1 impulse experiments. *τ_tank,FF,i_* was simultaneously regressed across all 4 mass flow rates, while also regressing *R_FF_*. Additionally, each value had a corresponding “goodness of fit—ε” value. In total, 9 feed frame parameters were regressed.

### 2.4. Analytical Methods

For tablets tested via HPLC, a composite sample of 10 tablets was evaluated for each sample point. Tablets were tested via HPLC to determine the average API concentration. Quantification was performed by ratio of peak areas to internal API standard. The HPLC assay employed was validated and demonstrated linearity from 50–150% of the target API concentration.

Similar to the method in [31], the API concentration in the blended powder was determined using an inline SentroPAT FO NIR (near-infra-red) spectrometer with InGaAs photodiode array detector in the 1100–2200 nm range equipped with a diffuse reflectance fiber optic probe (Sentronic GmbH). The NIR calibration consisted of 337 samples spanning approximately 80–150% of the target API concentration, with minimized correlation between any two components. A PLS (partial least-squares) model on the API was developed using calibration blend spectra and the composite assay of the API concentrations by HPLC (Agilent 110 equipped with quaternary pump and diode array detector) on the corresponding tablets as reference values in PLSToolbox (version 8.2.1) MATLAB (version 9.1.0.441655 (R2016b)) software. The PLS model using mean-centered Savitzky–Golay first derivative with 35 window and a second-order polynomial pre-treated spectra followed by standard normal variate normalization and mean centering had RMSECV (Root Mean Square Error of Cross-Validation) of 2.6% of the target API concentration and an R^2^ of 0.983.

## 3. Results 

### 3.1. Calculated Residence Time Distribution (RTD) Parameter Analysis

At each operating condition summarized in Figure 5, the *MRT* was calculated according to Equation (2) using the E(t) transformed data. As previously described, the mixing behavior in the Blender 1 impulse experiments contains the mixing behavior for both Blender 1 and Blender 2. Similarly, the feed frame response includes the mixing behavior of Blender 1, Blender 2, and the feed frame. The calculated *MRT* for Blender 1 and Blender 2 at each mass flow rate and blender speed combination is summarized in Table 1. Since the only operating parameter that was studied for the feed frame was mass flow rate, the calculated *MRT* for the feed frame is summarized only at each mass flow rate in Table 1.

It can be noted in Table 1 that the *MRT* for the 300 rpm Blender 2 conditions, across all mass flow rates, are relatively shorter compared to other conditions. This behavior is most likely due to an elongated tail relative to the rest of the data set. The elongated tail behavior is attributed to the high Blender 2 impeller speed, as the tracer experiments in the single blender at high speeds are very short. As shown in Equation (2), the integral of the t∗E(t) is taken to calculate the mean residence time. The elongated tail appears to skew the integral and reduce the calculated *MRT* further. 

**Figure 6:**
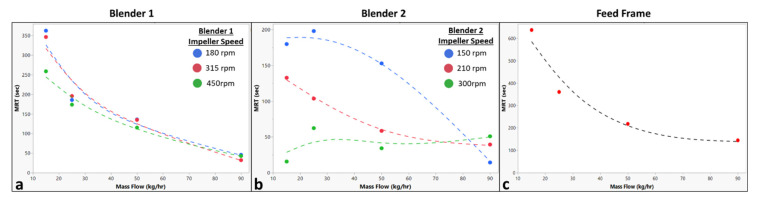
Plots of the calculated mean residence time for each blending unit. (**a**) Plot for the calculated Blender 1 mean residence time versus the total system mass flow rate with series at Blender 1 impeller speed. (**b**) Plot for the calculated Blender 2 mean residence time versus the total system mass flow rate with series at Blender 2 impeller speed. (**c**) Plot for the calculated feed frame mean residence time versus the total system mass flow rate.

The calculated mean residence time values summarized in Table 1 are shown in Figure 6 plotted against the corresponding operating conditions. Figure 6a shows a strong inverse relationship between Blender 1 *MRT* and mass flow rate. However, Figure 6a also shows very little relationship between Blender 1 *MRT* and impeller speed, except a slight inverse relationship. Similarly, Figure 6b shows an inverse relationship between Blender 2 *MRT* and mass flow rate, except at higher impeller speeds. Additionally, there appears to be a strong inverse relationship between Blender 2 *MRT* and impeller speed across all mass flow rates in Figure 6b. Figure 6c shows a strong inverse relationship between the feed frame *MRT* and mass flow rate.

### 3.2. Measured Residence Time Distribution (RTD) Parameter Analysis

Using the algorithm described in Section 2.3.1, the RTD parameters were optimized for each blending unit. The *R*_*B*1_ value and individual *τ*_*tank*,*B*1,*i*_ values were used to calculate the *MRT* in Blender 1 across the operating space summarized in Figure 5. The results from the regression are provided in Table 2. The goodness-of-fit values, ε, are also summarized in the table and provide an estimate of the model’s ability to predict the *MRT*. Within the same regression, the *R*_*B*1_ value was found to be 2.03 × 10^4^. As previously described, *R*_*B*1_ = *τ*_*tank*,*B*1,*i*_/*θ*_*B*1,*i*_ and is constant across all conditions for that blending unit. The relatively large ratio of *R*_*B*1_ indicates a much higher degree of mixing compared to a delay time within the blender. Thus, the mixing behavior in Blender 1 is best described as a well-back-mixed system rather than purely axial transport. This behavior is further discussed in Section 4.1.

The *R*_*B*2_ value and *τ*_*tank*,*B*2,*i*_ values were used to calculate the *MRT* in Blender 2 across the operating space summarized in Figure 5. The results from the regression are provided in Table 3, as well as the goodness-of-fit values. Within the same regression, the *R*_*B*2_ value was found to be 0.378. As previously described, $R_{B2}=\frac{\tau_{tank,B2,\;i}}{\theta_{B2,\;i}}$ and is constant across all conditions for that blending unit. The relatively small ratio of *R*_*B*2_ indicates a much higher delay time compared to mixing capacity within in the blender. Thus, the mixing behavior of Blender 2 is dictated by powder axial transport rather than a well-back-mixed system. The behavior agrees with the equipment set up, given that Blender 2 is set in an “all convective” position. This behavior is further discussed in Section 4.1.

The *R_FF_* value and *τ_tank,FF,i_* values at each condition were used to calculate the *MRT* in the tablet press across the operating space summarized in Figure 5. The results from the regression are provided in Table 4, as well as the goodness-of-fit values. The *R_FF_* value was found to be 0.389. As previously described, $R_{FF}=\frac{\tau_{tank,FF,\;i}}{\theta_{FF,\;i}}$ and is constant across all conditions for the unit. Similar to *R*_*B*2_, the relatively small ratio of *R_FF_* indicates a much higher delay time compared to mixing capacity within in the blender. The behavior agrees with the nature of the feed frame where a low degree of mixing is occurring, and the powder is merely transported into the tablet press. This behavior is further discussed in Section 4.1.

**Figure 7:**
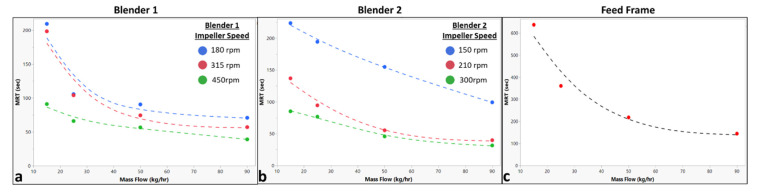
Plots of the measured mean residence time for each blending unit. (**a**) Plot for the measured Blender 1 mean residence time versus the total system mass flow rate with series at Blender 1 impeller speed. (**b**) Plot for the measured Blender 2 mean residence time versus the total system mass flow rate with series at Blender 2 impeller speed. (**c**) Plot for the measured feed frame mean residence time versus the total system mass flow rate.

The previously summarized measured mean residence time values are shown in Figure 7 and are plotted against the operating conditions. Figure 7a shows a strong inverse relationship between Blender 1 *MRT* and mass flow rate. Figure 7a also shows a slight inverse relationship between Blender 1 *MRT* and impeller speed, especially at higher mass flow rates. Similarly, Figure 7b shows a strong inverse relationship between Blender 2 *MRT* and mass flow rate. Additionally, Figure 7b shows a strong inverse relationship between Blender 2 *MRT* and impeller speed across all mass flow rates. Figure 7c shows a strong inverse relationship between the feed frame *MRT* and mass flow rate.

### 3.3. Comparison of Calculated and Measured Mean Residence Time (MRT)

**Figure 8:**
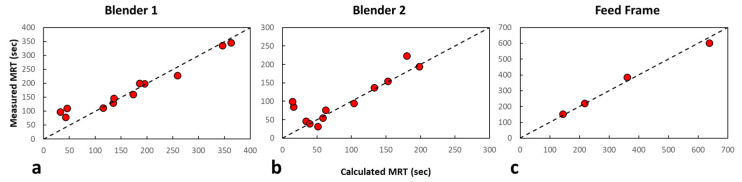
Parity plots for each blending unit comparing the different methods of determining the mean residence time. (**a**) Blender 1 parity plot of the calculated versus measured mean residence time. (**b**) Blender 2 parity plot of the calculated versus measured mean residence time. (**c**) Feed frame parity plot of the calculated versus measured mean residence time.

To assess the capability of the algorithm to calculate the *MRT*, both methods were compared to each other. Since the calculated Blender 1 and feed frame *MRT* included the other blending unit’s mixing behaviors, the appropriate measured responses were added together to produce a similar response to the calculated *MRT* for comparison purposes. As indicated in Figure 5, all Blender 1 experiments were conducted at the center-point Blender 2 impeller speed at each mass flow rate. Therefore, all Blender 1 measured *MRT* values were added with the center-point Blender 2 impeller speed *MRT* at each mass flow rate. Additionally, as indicated in Section 2.3, all feed frame experiments were conducted at the center-point Blender 1 and Blender 2 impeller speed at each mass flow rate. Therefore, all feed frame measured *MRT* values were added with the center-point Blender 1 and Blender 2 impeller speed *MRT* at each mass flow rate. The results of the comparison are shown in parity plots of each blending unit in Figure 8. The R2 values for Blender 1, Blender 2, and feed frame are 0.92, 0.98, and 1.00, respectively. These data indicate that the model algorithm is appropriately predicting the *MRT* of the data.

## 4. Discussion

### 4.1. Optimized RTD Prediction Curves

**Figure 9:**
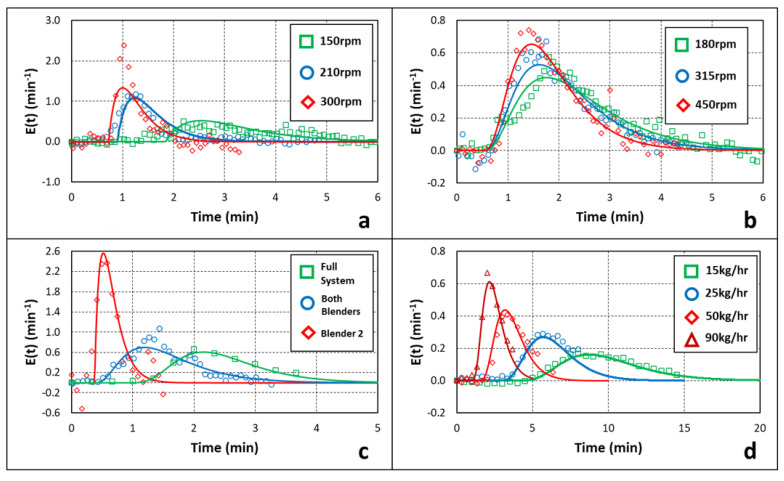
Impulse experimental results by mass flow. (**a**) Blender 2 pulses at 25 kg/h with varying Blender 2 impeller speed. (**b**) Blender 1 pulses at 50 kg/h with varying Blender 1 speed and constant Blender 2 speed. (**c**) Blender 2, Blender 1, and full-system pulses at 90 kg/h. (**d**) Full-system pulses at 15 kg/h, 25 kg/h, 50 kg/h, and 90 kg/h.

A subset of the resulting optimized curves is summarized in Figure 9. Figure 9a summarizes Blender 2 impulse experiments performed at 25 kg/h with varying Blender 2 impeller speeds. Figure 9b summarizes Blender 1 impulse experiments performed at 50 kg/h with varying Blender 1 impeller speeds and constant Blender 2 impeller speed. Figure 9c summarizes Blender 2, Blender 1, and full-system impulses performed at 90 kg/h with constant Blender 1 and Blender 2 impeller speeds. Figure 9d summarizes full-system impulses performed at 15 kg/h, 25 kg/h, 50 kg/h, and 90 kg/h. 

Figure 9a shows the transformed *E*(*t*) blend NIR data and corresponding model predictions for Blender 2 impulse experiments performed at 25 kg/h and 150 rpm, 210 rpm, and 300 rpm impeller speeds. The data indicate that there is a lengthening and broadening of the RTD response, suggesting an increase in residence time as Blender 2 impeller speed decreases, which is confirmed by the increase in *MRT* shown in Table 3. At the 300 rpm operating condition, the model prediction does not seem to capture the peak of the data. The transformed blend NIR data appear to have a 1-CSTR-like decay behavior. Capturing this type of blending behavior is a limitation of the equal-sized 2-CSTRmodel structure with decay. However, the curve appears to capture the data before and after the peak, therefore appropriately capturing the *MRT* of the data.

Figure 9b shows the transformed *E*(*t*) blend NIR data and corresponding model predictions for Blender 1 impulse experiments performed at 50 kg/h and 180 rpm, 315 rpm, and 450 rpm Blender 1 impeller speeds. Each impulse was conducted at a Blender 2 impeller speed of 210 rpm. The data indicate that there is a slight lengthening and broadening of the RTD response, suggesting there is also a slight increase in residence time as Blender 1 impeller speed decreases, which is confirmed by the increase in *MRT* shown in Table 2. 

Figure 9c shows the transformed *E*(*t*) blend NIR for Blender 1 and Blender 2 impulse experiments and transformed *E*(*t*) tablet HPLC results for full-system impulse experiments, with each corresponding model prediction. All impulse experiments were performed at 90 kg/h, 315 rpm Blender 1 impeller speed, and 210 rpm Blender 2 impeller speed. The figure indicates that there is a steep drop from the Blender 2 impulse curve to the Blender 1 impulse curve, where the Blender 1 impulse curve is inclusive of both blenders’ mixing behavior. The broader curve for the Blender 1 impulse experiments indicates that there is a higher degree of mixing occurring in Blender 1 than in Blender 2, which is confirmed by *R*_*B*1_ >> *R*_*B*2_. Since *R* = *τ_tank_*/*θ*, a larger value indicates the CSTR behavior dominates the mixing behavior of the blending unit. Additionally, there appears to be very little difference in delay time between the Blender 2 and Blender 1 curves. This further confirms that Blender 1 is dominated by a high degree of back-mixing and Blender 2 is dominated by the convective transport along the unit. The figure also indicates a delay between the Blender 1 impulse and the full-system data and only a small difference in the shape of the two curves. These data indicate there is a low degree of mixing occurring in the feed frame but an increased residence time due to the delay time, as shown in Table 4. This is further confirmed by the low *R_FF_* value, indicating the delay time caused by the hold-up inside of the transfer shoot is dominating the mixing behavior and residence time in the feed frame. 

Figure 9d shows the transformed *E*(*t*) tablet HPLC data for full-system impulse experiments at various mass flow rates with each corresponding model prediction. Impulse experiments were conducted at 15 kg/h, 25 kg/h, 50 kg/h, and 90 kg/h. All impulse experiments were conducted at 315 rpm Blender 1 impeller speed and 210 rpm Blender 2 impeller speed. The curves appear to flatten and broaden as the mass flow rate decreases. This behavior indicates that there is a higher degree of mixing as the mass flow rate decreases and, therefore, a higher residence time. This is confirmed in Table 2, Table 3 and Table 4, where the *MRT* increases as the mass flow rate decreases.

### 4.2. Empirical Relationship

As shown in Figure 7, there was an observed inverse relationship between the *MRT* versus the total system mass flow rate and impeller speed for both Blender 1 and Blender 2. Using the observed trends between the impeller speed, mass flow rate, and *MRT*, the relationship described in Equation (16) was developed and optimized for Blender 1 and Blender 2. The presented relationship is empirical in nature and intended to serve as a means to mathematically represent the impact of each process parameter of the mixing:

(16) $$MRT_{Blenders}=\frac{A}{\dot{m}}+\frac{B}{\beta }+\frac{C}{\dot{m}\cdot \beta }+D$$

where $MRT_{Blenders}$ is the mean residence time in seconds, $\dot{m}$ is the system mass flow in kg/h, β is the blender speed in rpm, and A, B, C, and D are constants specific to each blending unit (i.e., one set each for Blender 1 and Blender 2). 

As is also shown in Figure 7, there was an observed inverse relationship between the *MRT* and the total system mass flow rate for the tablet press feed frame. This inverse relationship can be described with Equation (17):

(17) $$MRT_{FeedFrame}=\frac{E}{{\dot{m}}^{2}}+D$$

where, $MRT_{FeedFrame}$ is the mean residence time in seconds, $\dot{m}$ is the system mass flow in kg/h, and E and D are constants specific to the feed frame.

**Figure 10:**
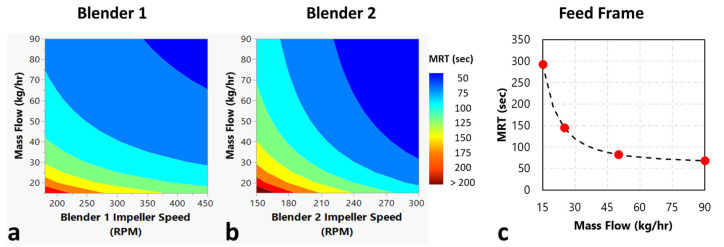
Plots describing the developed empirical relationship for each unit. (**a**) Contour plot for Blender 1 *MRT* versus mass flow rate and Blender 1 impeller speed. (**b**) Contour plot for Blender 2 *MRT* versus mass flow rate and Blender 2 impeller speed. (**c**) Plot for feed frame *MRT* versus mass flow rate.

**Figure 11:**
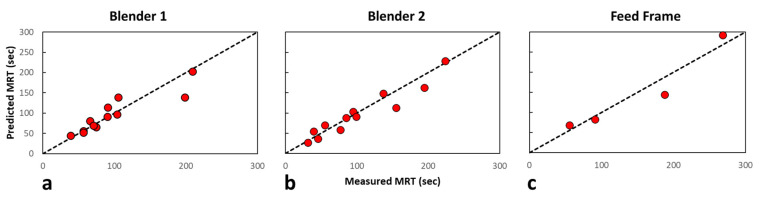
Parity plots for each blending unit comparing the measured and predicted mean residence time. (**a**) Blender 1 parity plot of the measured versus predicted mean residence time. (**b**) Blender 2 parity plot of the measured versus predicted mean residence time. (**c**) Feed frame parity plot of the measured versus predicted mean residence time.

A non-linear least-squares regression, developed in MATLAB, was used to estimate the constants for each blending unit using the *MRT* values and the corresponding operating conditions. The inverse of each goodness-of-fit value (ε) from the model optimization performed for each blending unit was used as a weighting factor in the regression. Therefore, measured *MRT* values with a larger ε value were weighted less than those with lower ε values. The individual constants for each blending unit are summarized in Table 5. This empirical relationship is depicted in Figure 10 for each unit, and the parity plot for each blending unit is described in Figure 11. The R2 values for Blender 1, Blender 2, and the feed frame are 0.95, 0.98, and 0.98, respectively, indicating a good correlation between the measured and predicted values.

### 4.3. Blending Capability

**Figure 12:**
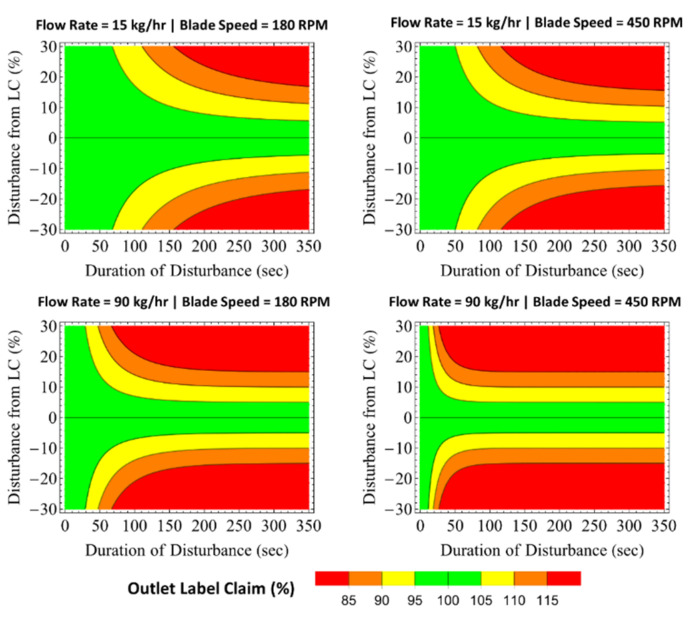
Funnel plots for Blender 1 at various flow rate and blade speed conditions.

Evaluation of blender dampability, or the capacity to reduce incoming disturbances, is represented using a funnel plot approach as described in recent publications [21]. Blender 1 predicted *MRT*s are used to calculate the regions of dampability of the funnels presented in Figure 12. The results show increases in mass flow rate and Blender 1 impeller speed have a negative impact on the dampability reduction. Thus, increasing the mass flow rate and impeller speed from 15 to 90 kg/h and 180 to 450 rpm significantly reduced to 95–105% the outlet target label claim (LC) region. For example, inlet disturbances of ±30% LC for a period of 50 s would be dampened to an outlet LC between 95 and 105% in the low-mass-flow-rate and slow-Blender-1-impeller-speed region of the plots. Similar disturbances in the high mass flow and fast impeller speed would lead to LC deviations at the outlet of more than ±15%, which can potentially yield product that does not meet release criteria.

**Figure 13:**
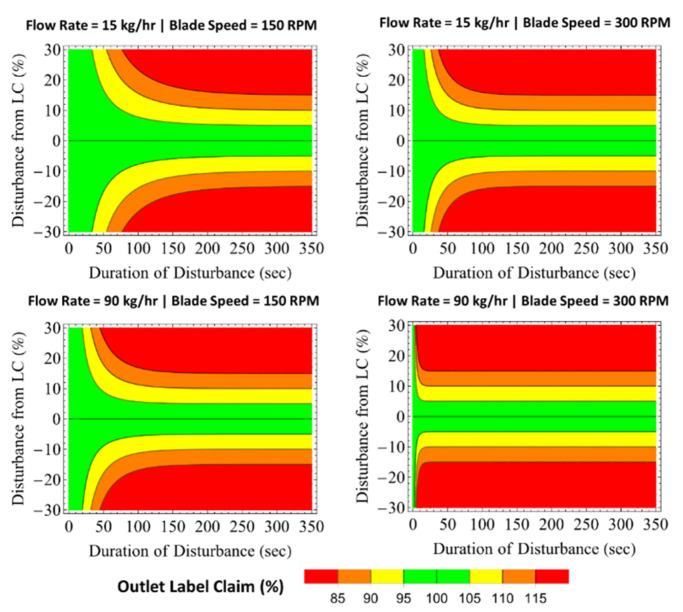
Funnel plots for Blender 2 at various flow rate and blade speed conditions.

Funnel plots for Blender 2 are shown in Figure 13. The funnel plot regions are diminished in comparison to Blender 1, particularly the high mass flow rate and fast impeller speed condition. In the 90 kg/h and 300 rpm condition, the blender’s dampability is expected to be minimal, with material effectively being solely transported axially in the unit without back-mixing.

**Figure 14:**
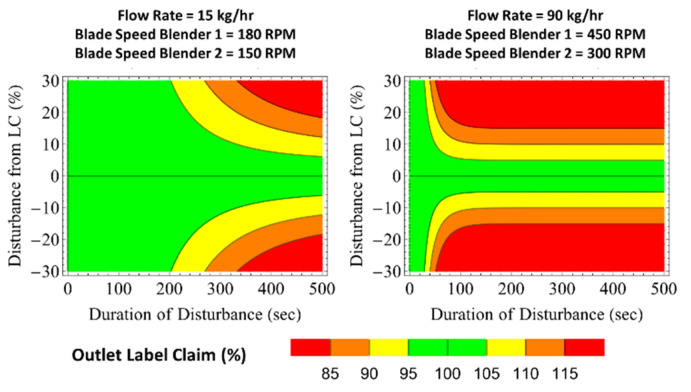
Funnel plots for Blender 1 + Blender 2 + feed frame at the extreme flow rate and blade speed conditions evaluated.

The funnel plots for the combined mixing systems (Blender 1 + Blender 2 + feed frame) at the two operational extremes of mass flow rate and both blender impeller speeds are shown in Figure 14. As expected, the low mass flow rate and slow impeller speeds lead to a significantly larger dampability region compared to the high flow and fast blade speed condition. Such results can be attributed to the extended back-mixing provided by additional time in the system. Most notably for the low mass flow rate and slow impeller speeds condition, input disturbances lasting up to 200 s spanning a range of ± 30% LC are expected to be dampened to be within 95–105% LC for the outgoing product. This result indicates that processes that are not well controlled at the feeder level may benefit from a reduction in both process conditions (i.e., flow rate and blade speeds). Nevertheless, it is important to consider that decreasing mass flow rate and impeller speed also increases the hold-up (i.e., residence mass) in the system, which in turn increases the amount of material that is not recoverable from the blenders and decreases the yield of product for the process.

### 4.4. Model Validation

The mathematical combination (i.e., convolution) of each of the blending units, at a specific set of operating conditions, combined with each individual LIW feeder output upstream, will provide a prediction of tablet API concentration downstream [21]. The incoming API concentration from the feeders, which is measured on a one second basis, is transformed to an API concentration of the tablets using a predefined set of RTD parameters for the given operating conditions. 

**Figure 15:**
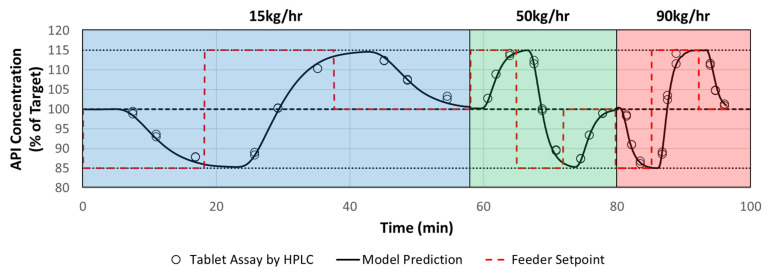
Model Validation Comparison of Tablet Assay to RTD Model Prediction.

Experiments were conducted to validate the ability of the RTD process model to predict tablet API concentration. Three sets of API concentration step change experiments were conducted at 15 kg/h, 50 kg/h, and 90 kg/h [22]. Positive and negative step changes in API concentration were conducted by increasing or decreasing the mass flow rate setpoint of the API, while inversely decreasing or increasing the mass flow rate setpoint of the other excipients, as to maintain a constant system mass flow. The step change sequence as a function of concentration is shown in Figure 15, where the target concentration varied from 85–115% of target concentration.

During each step change, tablet samples were collected at t10, t50, and t90, which correspond to the theoretical times for 10%, 50%, and 90% of the step change, respectively, to move through the system. Tablet samples were tested for composite assay in duplicate at each time point. The overall validation consisted of 54 samples, and the results of the validation are summarized in Figure 15.

**Figure 16:**
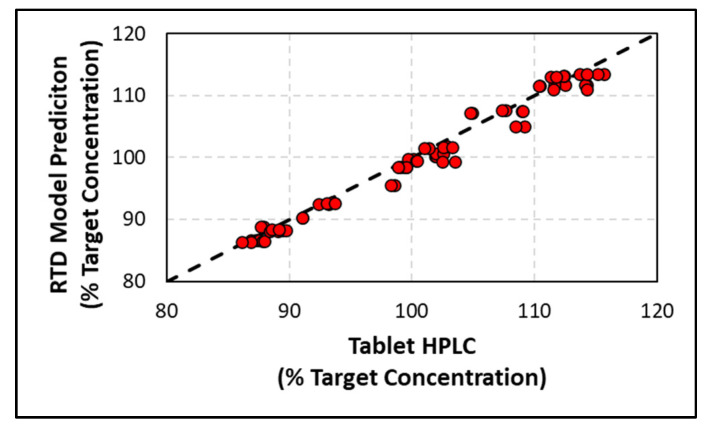
Parity plot for the model validation comparing the measured and predicted tablet concentration.

In order to compare the tablet assay results to the RTD model prediction, the root mean squared error of prediction (*RMSEP*) was used as described in Equation (18):

(18) $$RMSEP=\sqrt{\frac{\sum^{\;}{\left(x_{tablet}-x_{RTD\;Model}\right)}^{2}}{N}}$$

where *x_tablet_* is the concentration of API in tablets measured by HPLC, *x_RTD Model_* is the predicted concentration of API in tablets by the RTD model, and N is the number of samples tested (*N* = 54). The *RMSEP* calculated from the model validation summarized in Figure 15 was 1.4%. The predefined model validation acceptance criteria was 6%; therefore, the initial RTD model validation passed. A parity plot between the actual and predicted tablet concentrations for the steps evaluated in Figure 15 is shown in Figure 16 below, where the R2 value is 1.0, indicating that the model is predicting the tablet concentration very well.

**Figure 17:**
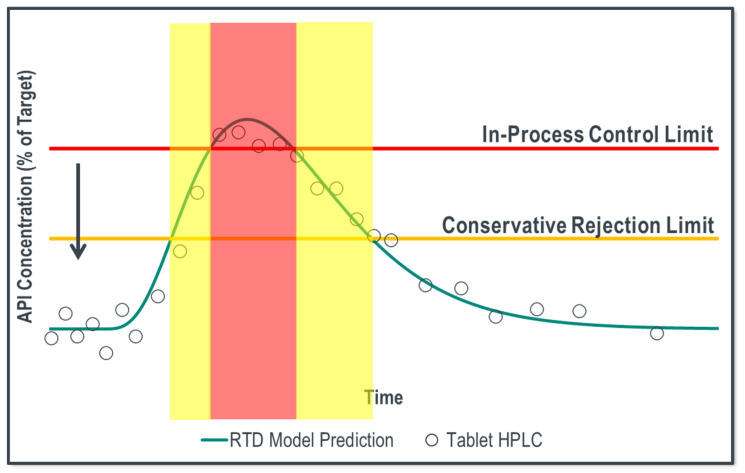
Graphical representation of implementing conservative rejection limits, where the red line is the product’s traditional in-process control limit and the yellow line is the calculated rejection limit.

The process model predicts tablet concentration in the core tablets and rejects potentially non-conforming material at the outlet of the tablet press. To ensure that individual tablet concentration remains between 85–115% of the target API concentration with 95% confidence, RTD rejection limits were developed using the validation acceptance criteria. These limits are defined in Equations (19) and (20):

(19) $$L_{lower}=85\%+Z_{0.95}\times \sqrt{RMSEP^{2}+\sigma_{weight}{}^{2}}$$

(20) $$L_{upper}=115\%-Z_{0.95}\times \sqrt{RMSEP^{2}+\sigma_{weight}{}^{2}}$$

where *Z*_0.95_ = 1.645 is the *z* score for a 95% one-sided confidence interval, *RMSEP* represents the variance of the RTD process model concentration prediction as defined by the root mean squared error of prediction acceptance criteria, which is used to represent the RTD process model RSD, and *σ_weight_* represents the individual tablet weight variance. A graphical representation of implementing these limits is summarized in Figure 17.

The red line represents the in-process control limit (e.g., 85% or 115%) and the yellow line represents the calculated limits using the validation acceptance criteria described in Equations (18) and (19). Traditionally, the RTD model would reject material within the red box; however, implementing tighter statistically developed limits, material is rejected starting at the yellow boxes. This ensures that non-conforming material is rejected from the process by incorporating sources of error during model development.

## 5. Conclusions

In these studies, an RTD-based process model was designed, developed, and validated for use in a continuous direct compression drug product control strategy. The methodology utilized a 2-CSTR-in-series model with a delay to estimate the mean residence time of different blending units within the process. In this work, studies were performed across various operating conditions for Blender 1 impeller speed, Blender 2 impeller speed, and total system mass flow rate, where the mean residence time was evaluated across the 20 different conditions studied.

Ultimately, strong correlations were observed between the operating conditions and the mean residence time, and an empirical equation was provided to relate the process input parameters to the output mean residence time. These equations were developed for the entire processing range and allowed independent predictions of mean residence time within the range studied, given a set of process input parameters.

The final part of these studies included validating the empirical equation developed. Multiple step changes were performed at mass flow rates within the operating range studied. Tablets collected at pre-determined time points throughout the step changes were tested for API concentration and compared to the model prediction. Through all 54 tablets tested, the root mean square error of prediction (*RMSEP*) was calculated to be 1.4%, ultimately passing the validation criteria. The successful validation allowed the RTD process model and related methodology to be incorporated into a successful commercial control strategy. This RTD control strategy involved statistically updating the model rejection limits to incorporate the different sources of error throughout the model development process. This overall strategy and this methodology were accepted by regulatory authorities.

## Figures and Tables

**Table 1:** Calculated *MRT* Using Equation (2) for Blender 1, Blender 2, and Feed Frame.

Mass Flow (kg/h)	Unit	Blade Speed (rpm)	*MRT* (s)
15	Blender 1	180	362.5
		315	346.4
		450	258.8
	Blender 2	150	179.8
		210	132.7
		300	15.60
	Feed Frame	-	362.5
25	Blender 1	180	185.6
		315	195.7
		450	173.7
	Blender 2	150	198.0
		210	103.6
		300	62.34
	Feed Frame	-	346.4
50	Blender 1	180	135.9
		315	134.8
		450	115.1
	Blender 2	150	152.8
		210	58.52
		300	34.16
	Feed Frame	-	258.8
90	Blender 1	180	45.57
		315	32.07
		450	42.73
	Blender 2	150	14.25
		210	39.36
		300	50.90
	Feed Frame	-	185.6

**Table 2:** Regressed Mean Residence Time Values and Goodness-of-Fit Values for Blender 1.

Mass Flow (kg/h)	Blade Speed (rpm)	*MRT* (s)	ε (s)
15	180	209	10.9
	315	198	6.10
	450	91.0	4.02
25	180	106	4.25
	315	104	2.26
	450	66.0	2.45
50	180	90.5	2.37
	315	74.5	1.34
	450	56.6	1.74
90	180	70.8	2.35
	315	57.0	1.07
	450	39.0	1.02

**Table 3:** Regressed Mean Residence Time Values and Goodness-of-Fit Values for Blender 2.

Mass Flow (kg/h)	Blade Speed (rpm)	*MRT* (s)	ε (s)
15	210	137	4.84
	150	224	12.6
	300	85.2	3.41
25	210	94.6	2.95
	150	194	10.3
	300	76.7	3.16
50	210	55.4	1.67
	150	155	6.13
	300	45.8	1.55
90	210	39.9	1.11
	150	99.3	4.17
	300	31.6	1.02

**Table 4:** Regressed Mean Residence Time Values and Goodness-of-Fit Values for the Feed Frame.

Mass Flow (kg/h)	*MRT* (s)	ε (s)
15	292.5	22.9
25	145.0	14.8
50	82.8	7.05
90	68.5	4.29

**Table 5:** Regressed Blender Constants for Each Blending Unit.

Unit	A (s-kg/h)	B (s-rpm)	C (s-kg-rpm/h)	D (s)	E (s-kg^2^/h^2^)
Blender 1	4.65 × 10^2^	3.54 × 10^3^	3.49 × 10^5^	23.2	-
Blender 2	−2.70 × 10^2^	1.48 × 10^4^	4.12 × 10^5^	−34.6	-
Feed Frame	-	-	-	62.1	5.19 × 10^4^

## Data Availability

Datasets used as a part of these studies are proprietary under Merck & Co., Inc. Redacted datasets are available upon request through the corresponding author.
